# Neuronal Subset-Specific Migration and Axonal Wiring Mechanisms in the Developing Midbrain Dopamine System

**DOI:** 10.3389/fnana.2017.00055

**Published:** 2017-07-10

**Authors:** Sara Brignani, R. J. Pasterkamp

**Affiliations:** Department of Translational Neuroscience, Brain Center Rudolf Magnus, University Medical Center UtrechtUtrecht, Netherlands

**Keywords:** midbrain dopamine system, development, substantia nigra, ventral tegmental area, neuronal subsets, striatum, axon guidance, migration

## Abstract

The midbrain dopamine (mDA) system is involved in the control of cognitive and motor behaviors, and is associated with several psychiatric and neurodegenerative diseases. mDA neurons receive diverse afferent inputs and establish efferent connections with many brain areas. Recent studies have unveiled a high level of molecular and cellular heterogeneity within the mDA system with specific subsets of mDA neurons displaying select molecular profiles and connectivity patterns. During mDA neuron development, molecular differences between mDA neuron subsets allow the establishment of subset-specific afferent and efferent connections and functional roles. In this review, we summarize and discuss recent work defining novel mDA neuron subsets based on specific molecular signatures. Then, molecular cues are highlighted that control mDA neuron migration during embryonic development and that facilitate the formation of selective patterns of efferent connections. The review focuses largely on studies that show differences in these mechanisms between different subsets of mDA neurons and for which *in vivo* data is available, and is concluded by a section that discusses open questions and provides directions for further research.

## Introduction

The dopamine system of the ventral midbrain (mDA system) can be subdivided into three main nuclei: substantia nigra pars compacta (SNc, A9), ventral tegmental area (VTA, A10), and retrorubral field (RRF, A8). Dopaminergic neurons of the mDA system are characterized by the synthesis and release of the neurotransmitter dopamine, and the expression of tyrosine hydroxylase (TH) and the dopamine transporter (DAT). SNc mDA neurons contribute to the control of voluntary movement and their selective degeneration is a pathological hallmark of Parkinson’s disease (Kalia and Lang, 2015). VTA mDA neurons play a role in positive and negative reinforcement, decision making, working memory, and aversion (for a review see, Morales and Margolis, 2017). Dopamine imbalance in VTA mDA neurons has been implicated in schizophrenia, attention deficit hyperactivity disorder (ADHD), obsessive-compulsive disorder (OCD), addiction, and depression (Winterer and Weinberger, 2004; Faraone et al., 2005; Chaudhury et al., 2012; Milton and Everitt, 2012). Their important physiological functions and implication in human disease has triggered an enormous interest in understanding the development and function of mDA neurons.

It is becoming clear that neurons within the anatomically defined SNc and VTA nuclei are not homogeneous. Rather multiple distinct mDA neuron subsets exist within and across the boundaries of the SNc and VTA. For example, subsets that differ by specific molecular markers, by afferent inputs, and by the brain structures they innervate. To understand how these differences arise, the developmental origin and molecular programs in mDA neuron subsets are studied intensively. It is likely, and in part known, that different mDA neuron subsets express specific molecular cues that allow subset-specific differentiation, migration and axon guidance.

In this review, we first summarize and discuss our current knowledge of the neuron subsets present in the mDA system. Then, molecular mechanisms are highlighted that aid mDA neurons in migrating to their final position in the midbrain and that allow the formation of selective patterns of efferent connections. We will focus mainly on studies that show differences in these mechanisms between different subsets of mDA neurons and for which *in vivo* data is available. For other studies on this topic which are not covered here we refer to other reviews (Van den Heuvel and Pasterkamp, 2008; Prestoz et al., 2012). The review is concluded by a section that discusses open questions and provides directions for further research.

## Neuronal Diversity in the mDA System

### Identification of mDA Neuron Subsets

**FIGURE 1:**
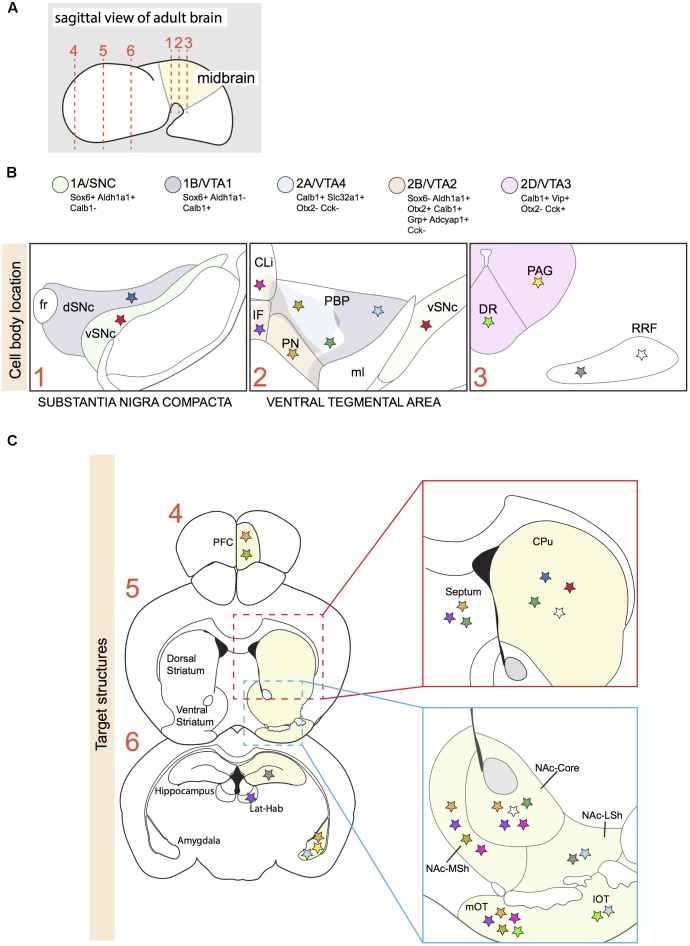
Projection areas of molecularly defined subsets of dopaminergic neurons in the adult brain. **(A)** Sagittal representation of an adult brain. Numbered dotted lines refer to coronal views in **(B,C)**. **(B)** Overlap of anatomically defined domains (dSNc, vSNc, PBP, PN, CLi, and IF) and mDA neuron clusters identified by specific molecular signatures (1A/SNC, 1B/VTA1, 2A/VTA4, 2B/VTA2, and 2D/VTA3; see **Figure 2**). Each cluster is defined by a few distinctive molecular markers and by unique colors. Colored stars represent mDA neuron subsets projecting to specific brain structures in C. **(C)** A colored star in C represents the brain region innervated by a specific mDA subset identified by a colored star in B. Stars with the same color represent mDA subsets and their target structures, respectively (Gasbarri et al., 1996; Ikemoto, 2007; Lammel et al., 2008; Matsuda et al., 2009; Stamatakis et al., 2013; Poulin et al., 2014; Aransay et al., 2015; Khan et al., 2017). vSNc, SNc ventral tier; dSNc, SNc dorsal tier; PBP, parabrachial pigmented nucleus; PN, paranigral nucleus; IF, interfascicular nucleus; CLi, caudal linear nucleus; DR, dorsal raphe nucleus; PAG, periaqueductal gray; RRF, retrorubral field; fr, fasciculus retroflexus; ml, medial lemniculus; PFC, medial prefrontal cortex; Lat-Hab, lateral habenula; CPu, caudate-putamen; NAc, nucleus accumbens; MSh, medial shell; LSh, lateral shell; mOT, medial olfactory tubercle; lOT, lateral olfactory tubercle.

Historically, anatomical and cytological features have been used to subdivide mDA neurons into subsets. According to this approach, SNc can be divided into a ventral and dorsal tier, whereas the VTA includes the parabrachial pigmented nucleus (PBP), the paranigral nucleus (PN), the caudal linear nucleus (CLi), the interfascicular nucleus (IF), and the rostral linear nucleus of the raphe (RLi) (Fu et al., 2012) (**Figures 1A,B**). Molecular markers exclusively expressed by single mDA subsets have not been identified yet. However, the expression of a few genes is commonly used to molecularly distinguish larger mDA domains. For example, the glycosylated active form of the dopamine transporter (glyco-DAT) and the G-protein-gated inwardly rectifying K^+^ channel (Girk2) are more abundantly expressed by SNc and dorso-lateral VTA mDA neurons (Schein et al., 1998; Thompson et al., 2005; Afonso-Oramas et al., 2009), while Calbindin 1 (Calb1) expression is enriched in mDA neurons of the VTA and of the dorsal tier of the SNc (Thompson et al., 2005; Di Salvio et al., 2010; Fu et al., 2012). Within the VTA, the transcription factor Otx2 strongly labels ventro-medial mDA neurons and gradually deceases in the central and dorso-lateral VTA (Simeone et al., 2011).

The development and use of single-cell RNA approaches has recently led to a further subdivision of the SNc and VTA on basis of molecular features (Poulin et al., 2014; La Manno et al., 2016). In one study, mDA neurons were collected at postnatal day 4 (P4) using a dopaminergic neuron-specific Cre-driver mouse line (*Slc6a3-Cre*) crossed with a TdTomato Cre-reporter mouse. qPCR was performed on single mDA neurons to determine expression levels of 96 selected genes. An unbiased coefficient similarity hierarchical clustering analysis allowed the clustering of cells in groups on basis of their gene expression profiles. This method identified two main cell clusters (cluster 1 and cluster 2). On basis of their expression profiles, mDA neurons from cluster 1 were more similar to SNc neurons, whereas neurons from cluster 2 had an expression profile related to VTA neurons. The two clusters were subsequently subdivided in two and four cell types, respectively (i.e., 1A, B, 2A-D) (Poulin et al., 2014). Another study analyzed P21 mDA neurons, collected using the same genetic strategy as described above, by single-cell RNA sequencing (RNAseq). In this second study, five cell types were identified (SNC, VTA1-4) (La Manno et al., 2016).

**FIGURE 2:**
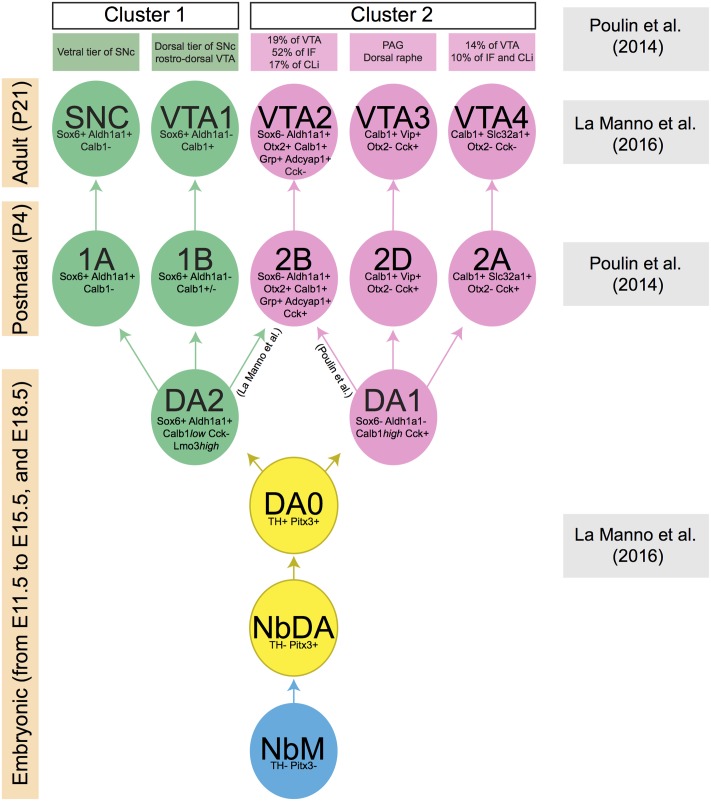
Lineage of mDA neuron subsets obtained by the comparison of two recent publications (Poulin et al., 2014; La Manno et al., 2016). Each mDA neuron cluster is defined by a few distinctive molecular markers which are indicated in the cluster. Boxes at the left indicate the developmental stage at which a specific collection of clusters is present (embryonic, postnatal, and adult phases). Boxes at the right indicate the publication that generated the data at specified time points. The green and pink boxes at the top list the anatomically defined domains in SNc or VTA in which the molecularly defined mDA clusters are positioned. During embryonic development, a group of mDA precursors (NbM, blue circle) gives rise to two consecutive groups of immature mDA subsets (NbDA and DA0, yellow circles). Then, two clusters of mature mDA neurons (DA1 and DA2, pink and green circles, respectively) originate from DA0. DA1 is the ancestor of all clusters represented in pink circles, whereas DA2 is the ancestor of all clusters represented in green circles. Data are not conclusive on the origin of mDA cluster 2B/VTA2, which may originate from DA1 or DA2. Calb1 is variably expressed by mDA neurons of cluster 1B (indicated in figure as Calb1+/–).

By using a set of marker genes (listed in different groups in **Figure 2**), we compared the five cell types reported by La Manno et al. (2016) with the six cell types identified at P4 by Poulin et al. (2014) (**Figure 2**). Interestingly, each of the five cell types identified at P21 has a corresponding cell type identified at P4. This suggests that the mDA system can be subdivided in (at least) five cellular subsets, each of which is characterized by a specific molecular signature. Moreover, the analysis indicates that these five neuronal subsets are already in place at P4. One cluster identified at P4 (2C) does not correspond to a cluster found at P21. This cluster expresses genes shared by all populations of cluster 2 (e.g., Calb1, Cck, and Slc17a6), but none of the markers that define specific cell types within cluster 2 [Slc32a1 (2A), Adcyap1 (2B), and Vip (2D)] (Poulin et al., 2014). This group of mDA neurons (2C) might be composed of neurons that are not completely mature at P4, and that acquire an adult phenotype at later stages, becoming part of one of the other subsets of cluster 2.

Comparison of the dopaminergic subnuclei as defined on basis of anatomical features with the location of the five subsets described above (SNc/1A, VTA1/1B, VTA2/2B, VTA3/2D, VTA4/2A) shows that cell types 1A and 1B mainly correspond to the ventral and dorsal parts of the SNc, respectively (**Figure 1B**). In Parkinson’s disease, mDA neurons of the ventral tier are known to be particularly vulnerable and in line with this cluster 1A is more selectively affected in a mouse model of Parkinson’s disease, as compared to cluster 2B (Poulin et al., 2014). The VTA contains neurons from different clusters (2A, 2B, 2C, and part of 1B) (**Figure 1B**). Cluster 2B is selectively positive for Grp and is positioned mainly in the PN and IF, which are nuclei with mDA neurons that project their axons to the NAc (Poulin et al., 2014; La Manno et al., 2016). In line with these results, an independent study demonstrated that VTA mDA neurons projecting to the NAc are Grp^+^ (Ekstrand et al., 2014). The most caudal VIP^+^ cluster (2D) is restricted to the dorsal raphe nucleus (DR) and periaqueductal gray (PAG) (Poulin et al., 2014; La Manno et al., 2016). Immunostaining for Vip, which is expressed by mDA axons, demonstrates that these mDA neurons establish connections with the stria terminalis and the amygdala (Poulin et al., 2014), indicating that molecular profiles are correlated with specific projection patterns. None of the identified cell-types is located in the RRF, even though the *Slc6a3-Cre* mouse line efficiently labels this structure (Bäckman et al., 2006). A possible explanation for this observation is that the RRF may host relatively small cell clusters which are not identified by the currently applied RNAseq and data analysis methods. It should also be noted that Poulin et al. (2014) and La Manno et al. (2016) used different techniques to identify subset-specific molecular profiles. It is therefore likely that in future studies additional subsets of mDA neurons are identified.

One of the studies described above also performed an unbiased analysis based on single-cell RNAseq data obtained from embryonic ventral midbrain tissue (collected from E11.5 to E15.5, and at E18.5). This procedure allowed the identification of a group of mDA precursors (medial neuroblasts, NbM), two immature mDA cell-types (NbDA and DA0), and two clusters of mature mDA neurons (DA1 and DA2) (La Manno et al., 2016). At E18.5, DA2 neurons express Aldh1a1, Sox6, and Calb1*low* which, in the adult brain, label three cell-subsets (1A/SNC, 1B/VTA1, 2B/VTA2). This suggests that DA2 neurons may be a common ancestor of these three groups (**Figure 2**), whereas DA1 may give rise to 2D/VTA3 and 2A/VTA4 (La Manno et al., 2016). However, it is important to note that according to the study of Poulin et al. (2014) the 2B/VTA2 subset belongs to cluster 2 and therefore it may derive from DA1 rather than DA2. Further work is needed to establish which ancestor (DA1 or DA2) generates the mDA subset 2B/VTA2. Interestingly, previous work has shown that the differentiation of the Calb1^+^/Aldh1a1^+^ mDA neurons (which correspond to cluster 2B/VTA2) requires the expression of the transcription factor Otx2. The deletion of *Otx2* gene from the midbrain at early developmental time points causes the depletion of the large majority of Calb1^+^ and Aldh1a1^+^ mDA neurons of the VTA, with only the mDA neurons of the dorso-lateral VTA (Grik2^+^) surviving into adulthood (Di Giovannantonio et al., 2013).

The identification of mDA neuron subsets, their developmental origin and molecular signatures is important for understanding how subset-specific connectivity is established in the mDA system. For example, defining clusters of mDA precursors, and immature and mature mDA neurons (NbM, NbDA, DA0, DA1, and DA2) from the RNA expression analysis allows the identification of guidance genes expressed by these neuronal populations (**Table 1**) (La Manno et al., 2016). Members of different guidance cue families (e.g., Semaphorins, Ephrins) can be detected in different clusters. Guidance genes expressed at early developmental time points might play a role in the migration and/or axon guidance of mDA neuron subsets. In the following sections, we discuss what is currently known about the mechanisms that control mDA neuron migration and axon guidance, with a particular emphasis on subset-specific mechanisms.

**Table 1:** Axon guidance genes expressed by dopaminergic precursors, and immature and mature mDA neurons.

NbM	NbDA	DA0	DA1	DA2
*DCC*	*DCC*	*DCC*	*DCC*	*DSCAM*
*Draxin*	*Draxin*	*Draxin*	*Draxin*	*Sema4D*
*Sema6C*	*Sema6C*	*Sema3F*		*EphA5*
*PlxnB1*	*Nrp2*	*Sema5B*		
*Nrp2*	*EphA5*	*PlxnC1*		
*EphA3*	*EfnB1*	*EfnB2*		

*mRNA expression of axon guidance genes in mDA precursors (medial neuroblasts, NbM), two immature mDA cell-types (NbDA and DA0), and two clusters of mature mDA neurons (DA1 and DA2). These molecules might be involved in migration and/or axon guidance of mDA neuron subsets. Data are obtained from Linnarsson’s lab webpage (www.linnarssonlab.org/ventralmidbrain) (see **Figure 2**).*

## Cellular and Molecular Mechanisms of mDA Neuron Migration

### Distinct Origins and Migratory Routes of SNc and VTA mDA Neurons

**FIGURE 3:**
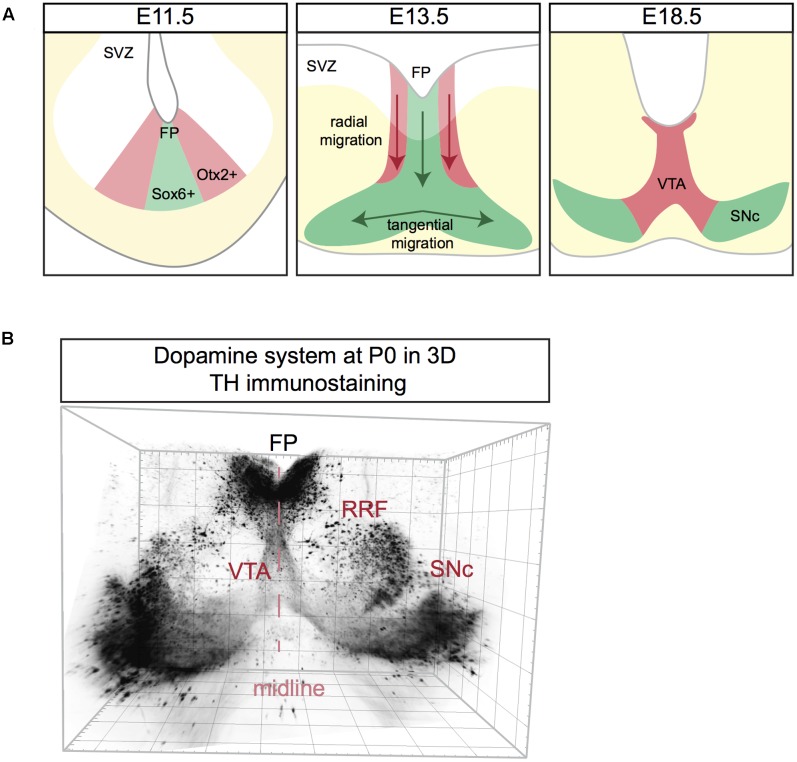
Schematic representation of mDA neuron migration **(A)** and a 3D reconstruction of the dopamine system at postnatal day (P) 0. **(A)** mDA neurons are generated in the subventricular zone (SVZ). SNc mDA progenitors are Sox6^+^ (green) and are positioned in the medial floor plate (FP). VTA mDA progenitors are Otx2^+^ (red) and are located in the lateral FP. First, mDA neurons migrate radially aligned along radial glia fibers. Next, only SNc mDA neurons migrate tangentially probably by using contralateral axons as a scaffold. At E18.5 mDA neuron migration is complete. **(B)** Coronal view of a 3D reconstruction of a P0 mouse brain immunostained using an anti-TH antibody (Brignani and Pasterkamp, unpublished data). Tissue was cleared using 3DISCO and imaged using Ultramicroscope lightsheet microscopy. The mDA system extends in three dimensions, with the neurogenic FP positioned caudally to the two wings of SNc. For other 3D views of the mDA system, see **Figure 4**.

In mice, mDA neurons are born between E10.5 and E14.5. The majority of SNc mDA neurons is born around E10.5, while neurogenesis of VTA mDA neurons is delayed and peaks around E11.5 (Bayer et al., 1995; Bye et al., 2012). Both neuron populations originate from progenitor cells positioned in the floor plate (FP) of the midbrain ventricular zone (VZ). mDA progenitors are divided into two subsets in the medial and the lateral zones of the FP. Around E8.5-E9.5, Sonic hedgehog (Shh) is exclusively expressed by medial mDA progenitors. However, at E11.5 Shh is no longer expressed in the medial zone, but is restricted to the lateral zone (Joksimovic et al., 2009; Blaess et al., 2011). This specific spatiotemporal pattern of Shh expression was recently exploited to perform fate mapping of mDA neurons and revealed that medial progenitors give rise to SNc mDA neurons, while lateral progenitors generate VTA mDA neurons (Blaess et al., 2011; Bodea et al., 2014). This observation was confirmed via immunostaining for Sox6 and Otx2/Nolz1, markers for both progenitors and mature mDA neurons of SNc and VTA, respectively. Sox6^+^/Otx2*^weak^*/Nolz1^-^ progenitors are located to the medial zone of the FP and Sox6^-^/Otx2*^strong^*/Nolz1^+^ progenitors are confined to the lateral zone (Panman et al., 2014) (**Figure 3A**).

Following neurogenesis, post-mitotic mDA migrate from the FP to their final destination in the marginal zone (MZ). This migratory stage is characterized by two phases. During the first phase SNc and VTA mDA neurons undergo radial migration. Migrating VTA neurons (Otx2^+^/Nolz1^+^) originating from lateral progenitors are positioned laterally in the intermediate zone (IZ), while SNc neurons (Sox6^+^) generated from medial progenitors are located medially in the IZ (Panman et al., 2014) (**Figure 3A**). Both VTA and SNc neurons show leading and trailing processes that are oriented radially, aligned with the fibers of radial glia-like (RGL) cells (Shults et al., 1990; Bodea et al., 2014). RGL fibers are thought to act as scaffolds for radially migrating mDA neurons, similar to the role of these cells in other regions of the developing nervous system (Marín et al., 2010). During the second phase of mDA neuron migration, SNc mDA neurons migrate tangentially, which results in their movement from the medial toward the lateral MZ. This ultimately results in the formation of the characteristic wing-like SNc structure observed in the postnatal and adult brain (**Figure 3A**). It has been proposed that during tangential migration the leading processes of SNc mDA neurons follow tangential fibers, presumably axons originating from neurons positioned in the lateral midbrain (Kawano et al., 1995). The leading and trailing processes of VTA mDA neurons are almost exclusively oriented radially, and not tangentially. Therefore, it is thought that VTA neurons migrate mainly radially (Bodea et al., 2014).

The dopamine system is an extremely complex structure that extends in three dimensions, with the neurogenic FP positioned more medio-caudally than the two wings of the developing SNc, which are protruding toward the rostro-lateral edges of the midbrain (**Figure 3B**). Data on the migration of mDA neurons along the anterior–posterior (A-P) axis is lacking and most of the studies on mDA neuron migration are performed on coronal sections at intermediate A-P levels of the mDA system. It is likely that the development of the mDA system not only involves radial and tangential migration of mDA neurons but also movement along the A-P axis.

### Guidance Molecules Involved in Radial Migration of mDA Neurons

Radial migration of post-mitotic mDA neurons starts in the FP, continues in the IZ and concludes in the MZ. During this migratory process, post-mitotic cells differentiate and become mDA neurons. RGL cells are positioned in the ventricular zone, both in the FP and basal plate (BP), and extend their fibers to the pial surface. Recent work identifies three distinct RGL cell types in the midbrain ventricular zone: RGL1, present in both the FP and BP; RGL2, confined to the BP; and RGL3, restricted to the FP. RGL1 and RGL2 are found from E11.5 to E15.5 during radial migration of mDA neurons. RGL3 is only detected at E15.5 (La Manno et al., 2016). The functional role(s) of these RGL cells in the midbrain remains unknown. However, single-cell RNAseq data of the ventral midbrain shows that several axon guidance genes are expressed by the three types of RGL cells (La Manno et al., 2016) (**Table 2**). These axon guidance genes may be involved in the development of RGL cell fibers but also in guiding migrating mDA neurons toward the pial surface. Interestingly, while several axon guidance genes are expressed in all three RGL cell types, others are specific to one or two subtypes. Further work is needed to determine the role of the three RGL cell types and of the specific axon guidance molecules they express. However, it is tempting to speculate that specific RGL cells and their associated cues may be responsible for guiding different subsets of mDA neurons.

**Table 2:** Canonical axon guidance genes expressed in three types of radial glia-like cells.

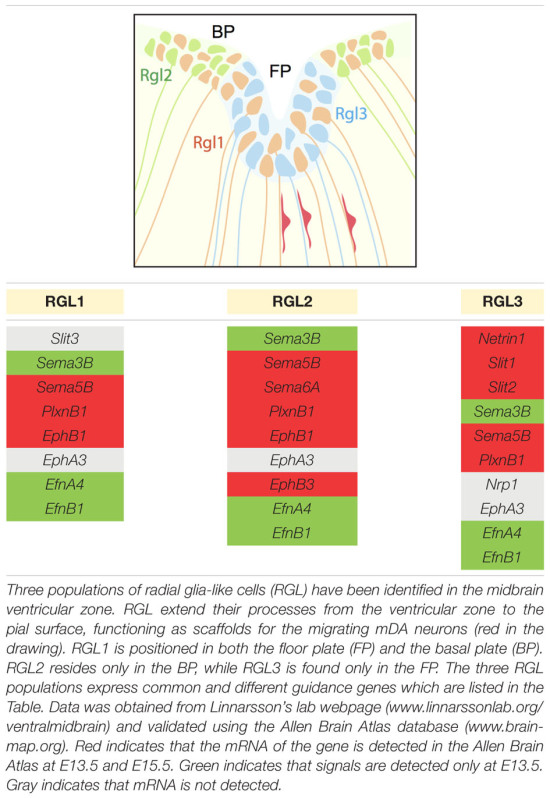

Although the specific RGL cell-derived molecules that regulate radial migration remain to be determined, meninges-derived chemokine signals have recently been shown to direct mDA neurons to the pial surface. Radially migrating mDA neurons express the chemokine receptor Cxcr4 from E10.5 to E14.5. Cxcr4 is a G protein-coupled receptor that is activated by the chemokine Cxcl12, which induces the phosphorylation of its C-terminal domain. Cxcl12 mRNA is expressed by meningeal cells during the period of mDA neuron migration (Yang et al., 2013; Bodea et al., 2014). *In vitro* meningeal explants attract migrating mDA neurons. This effect is blocked by administration of a Cxcr4 antagonist (Yang et al., 2013). Ectopic expression of Cxcl12 in the reticular formation *in vivo* causes mDA neurons to follow an abnormal route away from the midline in the IZ toward the ectopic Cxcl12^+^ cells (Yang et al., 2013). The speed and trajectory of migrating mDA neurons are intact when Cxcr4/Cxcl12 signaling is perturbed *in vitro*, indicating that other aspects of migration are controlled by this chemokine system (Bodea et al., 2014). Analysis of *Cxcr4* and *Cxcl12* KO mouse brains reveals that lack of Cxcr4/Cxcl12 signaling *in vivo* causes an accumulation of mDA neurons in the IZ, in contrast to control conditions where these neurons reach the MZ (Bodea et al., 2014). Moreover, during the initial stage of radial migration (E11.5), mDA neurons lacking Cxcr4 have processes oriented in both radial and tangential directions, whereas most processes from neurons in wild-type mice show a radial orientation (Yang et al., 2013). At later developmental stages, defects are much milder in *Cxcr4* KO mice and absent in *Cxcl12* KO mice. This observation may hint at compensation of loss of chemokine signaling by other molecular signals (Yang et al., 2013; Bodea et al., 2014).

The cell surface receptor DCC and its ligand Netrin1 also play a role in mDA neuron migration. DCC protein is expressed by mDA neurons during the period of their migration (Xu et al., 2010). The Netrin1 promoter is active in the ventral midbrain, both in the FP and in TH^+^ nuclei (Li et al., 2014). *DCC* and *Netrin1* KO mice show ectopically (dorsally) positioned mDA neurons at P0, when mDA neuron migration has been completed (Xu et al., 2010; Li et al., 2014). Although the migration defects detected in *DCC* and *Netrin1* KO mice are similar, how DCC-Netrin1 signaling controls mDA neuron migration remains unknown. Interestingly, Netrin1 is expressed by RGL cells in the FP (**Table 2**) and it is therefore tempting to speculate that RGL cell-derived Netrin1 provides guidance for DCC^+^ mDA neurons during their radial migration.

### Guidance Molecules Involved in Tangential Migration of mDA Neurons

Following their radial migration, SNc mDA neurons migrate tangentially, in part under control of Reelin and its receptors. Reelin controls the correct layering and polarization of different brain areas, such as the cortex and hippocampus (D’Arcangelo, 2014; Förster, 2014). It is a component of the extracellular matrix and signals via two receptors called low density lipoprotein receptor-related protein 8 (Apoer2) and very low density lipoprotein receptor (Vldlr). Binding of reelin to its receptors induces tyrosine phosphorylation of the intracellular signaling protein Dab1.

In the *reeler* mouse, an autosomal-recessive mouse mutant carrying a disrupted *reelin* gene, the SNc does not extend laterally at intermediate A-P levels and mDA neurons accumulate in the VTA region. At rostral levels, the SNc develops normally and no significant change in the total number of mDA neurons is observed. *Dab1* deficient mice (*yotari* mice) and double KO mice for *Apoer2* and *Vldlr* show similar mDA neuron migration defects (Nishikawa et al., 2003; Kang et al., 2010; Bodea et al., 2014). This suggests that both receptors and Dab1 are mediators of reelin signaling in the midbrain. In line with these observations, both *in vivo* and *in vitro* experiments show that a subset of SNc neurons fails to orient their processes tangentially when reelin signaling is perturbed, while VTA mDA neurons are unaffected (Bodea et al., 2014). Reelin deficiency may alter the guidance scaffolds required for mDA neuron migration, but there is a lack of consensus on this hypothesis. Some studies report that tangential axons are intact in *reeler* mice at E15.5, whearas other work describes defects in the development of tangential axons in *reeler* and *yotari* mice as early as E14.5 (Nishikawa et al., 2003; Kang et al., 2010).

At E13.5, when mDA neurons are migrating both radially and tangentially, *reelin* mRNA is expressed by the red nucleus and absent from mDA neurons (Bodea et al., 2014). If the red nucleus is the main source of reelin for migrating mDA neurons, lack of red nucleus cells should cause malformation of the SNc. Interestingly, in *Nkx6-1* KO mice the number of cells in the red nucleus is dramatically decreased at E12.5, and at E18.5 only 27% of these neurons remain (Prakash et al., 2009). Nkx6-1 is a transcription factor expressed in the BP by progenitors of red nucleus neurons, but not by mDA progenitors nor by mDA neurons. In *Nkx6-1* KO mice, the dopamine system develops normally, suggesting that reelin expressed by the red nucleus may not be responsible for the correct tangential migration of SNc mDA neurons. Recently, reelin protein was detected in mDA neurons and in the surrounding area at P0, but not at earlier developmental stages (Sharaf et al., 2015). However, at P0 migration of mDA neurons is complete. In contrast, other work detects reelin in the extracellular space surrounding mDA neurons as early as E15.5. It is possible that other cells, such as embryonic striatal neurons which are reelin^+^ at E15.5, supply the midbrain with reelin through their axonal projections (Nishikawa et al., 2003). In this case, reelin may function as a chemoattractant for tangentially migrating SNc mDA neurons, and lack of reelin could prevent SNc mDA neurons from orienting their processes in the correct direction.

In conclusion, experimental data show that reelin and its receptors are necessary for the correct tangential migration of a subset of SNc mDA neurons. It remains to be determined whether reelin influences SNc mDA neuron movement directly by acting as an attractant guidance cue, or whether it is required for the correct development of the guidance scaffolds used by these neurons for migration.

## Growth and Guidance of mDA Axons

### mDA Efferent and Afferent Connections

**FIGURE 4:**
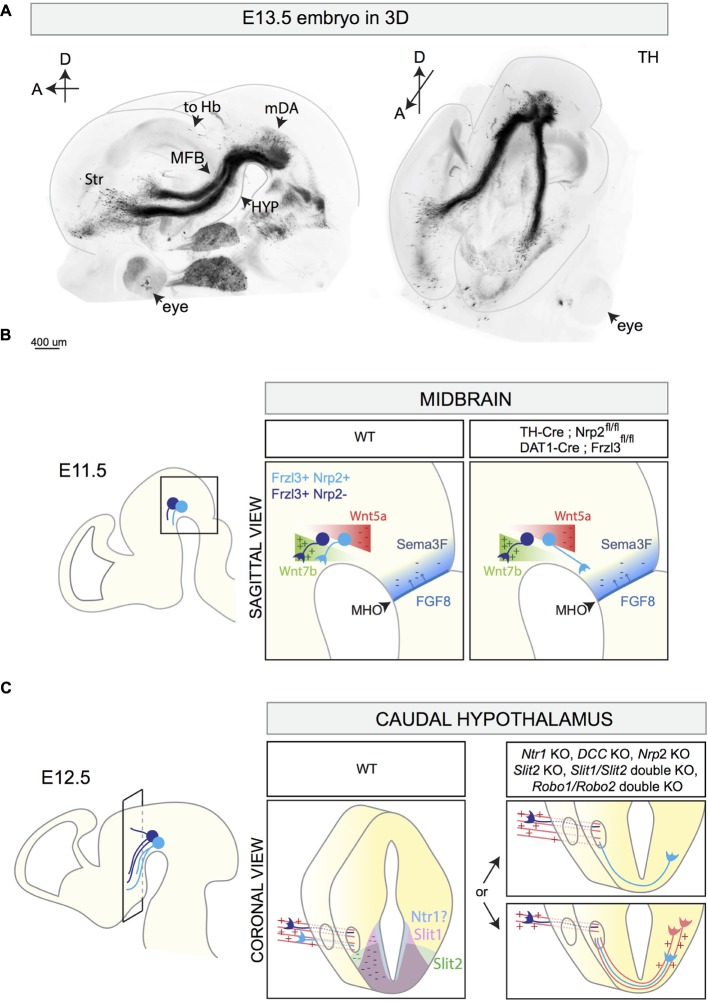
3D reconstruction of the E13.5 mDA system and schematic representations of different stages of mDA axon growth and guidance. **(A)** 3D reconstruction of a E13.5 mouse embryo head with a sagittal (left) and horizontal (right) view. The brain is immunostained for TH and cleared using the 3DISCO protocol (Brignani and Pasterkamp, unpublished data). 3D reconstruction shows mDA neurons positioned on top of the midbrain flexure. The majority of mDA axons elongates toward the forebrain forming the MFB. mDA axons projecting to the habenula (to Hb) do not grow in the MFB, but are instead oriented in a rostro-dorsal direction. At E13.5, the first mDA axons reach the striatum (Str). **(B)** The rostral orientation of mDA axons (represented in blue) in the midbrain is determined by the expression of gradients of guidance cues: Wnt7b acts as attractant (+) for growing mDA axons and shows an increasing caudo-rostral gradient, whereas Wnt5a functions as a repellent (–) and shows a rostro-caudal gradient. mDA axons express the receptor Fzd3 which mediates Wnt signaling. The conditional removal of *Fzd3* from mDA neurons causes the misrouting of a subset of mDA axons toward the caudal midbrain. Moreover, in normal conditions, neurons of the MHO secrete FGF8 which in turn induces the expression of the repellent Sema3F (–). The Sema3F receptor Nrp2 is expressed by a subset of medial mDA neurons. The conditional removal of Nrp2 from mDA neurons causes the caudal misrouting of a subset of mDA axons. **(C)** The caudal hypothalamus expresses *Netrin1*, *Slit1*, and *Slit2*. mDA axons elongate longitudinally most likely following other pre-existing axons that function as scaffolds (red parallel lines). In *Netrin1* KO, *DCC* KO, *Nrp2* KO, *Slit2* KO, *Slit1/Slit2* double KO, and in *Robo1/Robo2* double KO, a subset of mDA axons exits the MFB, turns ventrally, and crosses the midline growing to the contralateral side of the brain. This phenotype might be a result of lack of expression of repellent guidance cues (Netrin1, Slit1, and Slit2) or of the misrouting of pioneer axons which normally function as a scaffold for mDA axons. MFB, medial forebrain bundle; Str, striatum; HYP, hypothalamus; to Hb, indicates mDA fibers growing toward the habenula; MHO, midbrain–hindbrain organizer.

The mouse mDA system contains around 30.000 neurons and despite its small size it is connected to many brain areas (**Figures 1C**, **4A**). The majority of mDA axons is oriented rostro-ventrally in the diencephalon, and is tightly fasciculated into two ipsilateral axon bundles called the medial forebrain bundles (MFBs). The MFBs pass first through the ventral diencephalon and then toward the telencephalon. mDA axons projecting to the habenula do not elongate inside the MFB, but are instead oriented in a rostro-dorsal direction toward the lateral habenula, which resides in the diencephalon, using the fasciculus retroflexus as scaffold (Schmidt et al., 2014). Recent advances in brain tissue clearing and 3D reconstruction of axonal tracts has further unveiled the complex axon projections that derive from the mDA system (Belle et al., 2014; Renier et al., 2014) (**Figure 4A**). This approach holds great promise for analyzing the normal development of mDA axon connections and of defects in axon growth and guidance present in KO mouse models.

SNc and VTA mDA neurons target partly distinct areas in the forebrain. The SNc and the dorso-lateral VTA project to the dorsal striatum, forming the mesostriatal pathway. Medial SNc mDA neurons innervate the dorso-medial striatum, while lateral SNc mDA neurons project to the dorso-lateral striatum (**Figure 1C**) (Lerner et al., 2015). Within the striatum, each SNc mDA neuron generates extensive axonal arborizations, establishing connections with on average 75.000 striatal neurons, positioned in both the striatal patch and matrix structure (Matsuda et al., 2009), which are two striatal compartments defined by different biochemical markers and different afferent and efferent connections (Gerfen, 1992).

VTA mDA neurons mainly project to the prefrontal cortex (PFC), the amygdala, and the ventral striatum, which is subdivided in the medial and lateral olfactory tubercle (mOT and lOT, respectively) and the nucleus accumbens (NAc – core, medial shell, lateral shell), forming the mesocorticolimbic pathway (Ikemoto, 2007; Lammel et al., 2008). VTA mDA neurons projecting to more medial regions of the ventral striatum are distributed medially within the VTA, whereas laterally positioned VTA mDA neurons establish connections with more lateral nuclei (**Figure 1C**) (Ikemoto, 2007; Lammel et al., 2008; Beier et al., 2015). In contrast to SNc mDA neurons, which have few collaterals outside the striatum, single VTA mDA neurons can have axon collaterals projecting to different brain areas. For example, a mDA neuron in the PBP can innervate both the cortex and the amygdala, while a neuron in the PN can establish connections with the amygdala, the NAc-core, and the septum (Aransay et al., 2015). Other VTA mDA neurons, like those projecting to the lateral habenula, are more selective and do not have axon collaterals (Stamatakis et al., 2013).

SNc and VTA mDA neurons do not only differ with respect to the targets they innervate. Several studies in the past decade have also unveiled striking differences in the afferent inputs of SNc and VTA mDA neurons. For example, ventral striatum mainly innervates VTA mDA neurons, whereas dorsal striatum establishes connections with SNc mDA neurons. Interestingly, in the dorsal striatum, striatal neurons residing in striosomes establish connections preferentially with SNc mDA neurons, while striatal neurons of the matrix show a preference for GABAergic neurons located in the SN reticulata (Gerfen et al., 1987; Fujiyama et al., 2011; Watabe-Uchida et al., 2012). Axonal projections from the NAc to the ventral midbrain mainly target VTA mDA neurons. Furthermore, SNc mDA neurons receive inputs from the somatosensory and motor cortex, subthalamic nucleus, superior colliculus, PAG, and DR (Watabe-Uchida et al., 2012). VTA mDA neurons receive glutamatergic inputs from the medial PFC, pedunculopontine tegmentum, laterodorsal tegmentum nucleus, lateral habenula, PAG, bed nucleus of the stria terminalis, and DR. VTA mDA neurons receive GABAergic inputs from the rostromedial mesopontine tegmental nucleus, PAG, lateral hypothalamus, ventral pallidum. There are also local glutamate and GABA synapses onto VTA mDA neurons arising from neurons within the VTA (recently reviewed in Morales and Margolis, 2017).

In conclusion, the efferent and afferent connections of SNc and VTA mDA neurons are strikingly distinct. In the following section, we discuss the different stages of mDA axon guidance and the molecules involved. We highlight differences between mDA axonal subsets, cell subtype-specific expression of axon guidance receptors, and the differential response to guidance cues.

### Rostrally Oriented Axon Growth in the Midbrain

The first mDA axons appear around E11 and their rostral orientation is determined by the expression of gradients of axon guidance cues along the A-P axis. An important signaling center involved in the generation of these molecular gradients is the midbrain–hindbrain organizer (MHO), which is positioned at the boundary between the caudal midbrain and the rostral hindbrain (Raible and Brand, 2004). Several secreted proteins are expressed by MHO cells, including members of the wingless (Wnt) and the fibroblast growth factor (FGF) families. The secretion of FGF8 by the MHO induces the expression of the axon repellent Semaphorin3F (Sema3F) (Yamauchi et al., 2009). At early developmental time points, Sema3F inhibits mDA axon outgrowth *in vitro* via its receptor Nrp2, which is expressed by a subset of medially positioned mDA neurons (Hernández-Montiel et al., 2008; Kolk et al., 2009; Yamauchi et al., 2009). *Nrp2* KO mice display mDA axons aberrantly growing caudally toward the MHO (Yamauchi et al., 2009). A similar phenotype is detected when Nrp2 is conditionally removed from mDA neurons (using *TH-Cre* mice) (Kolk et al., 2009). Together, these results indicate that Sema3F creates a non-permissive territory in the caudal midbrain for early Nrp2^+^ mDA axons (**Figure 4B**).

Wnts also contribute to the rostral orientation of mdDA axons. Wnt5a is expressed in a caudal-high-to-rostral-low gradient in the midbrain, whereas Wnt7b is expressed in an opposite molecular gradient. mDA neurons express the core planar cell polarity (PCP) components Frizzled3, Celsr3, and Vangl2, necessary for transducing Wnt-mediated signaling. *In vitro*, both SNc and VTA axons are repelled by Wnt5a (Fenstermaker et al., 2010; Blakely et al., 2013) and attracted by Wnt7b (Fenstermaker et al., 2010; Fernando et al., 2014). These effects are mediated by the receptor Frizzled3 (Fenstermaker et al., 2010). In *Frizzled3*, *Celsr3*, and *Vangl2* KO mice, mDA axons lose their rostral orientation and display aberrant dorsal and caudal projections (Fenstermaker et al., 2010). Conditional deletion of Frizzled3 from mDA neurons (using *DAT-Cre* mice) causes similar defects in mDA axon orientation, with mDA axons forming a caudal tract that descends into the spinal cord (Hua et al., 2014). Abnormal, but transient, caudal mdDA axon projections are detected in *Wnt5a* KO mice (Fenstermaker et al., 2010). Together, these data show that Wnt-PCP signaling along the A-P axis of the midbrain is required for orienting mDA axons rostrally (**Figure 4B**).

### Axon Fasciculation in the MFB and Ipsilateral Projections

In the ventral diencephalon, mDA axons form the MFB that traverses the diencephalon toward its targets in the telencephalon (**Figure 4A**). Axon guidance receptors and cues play a role in the fasciculation of axons within the MFBs and in preventing mDA axons from crossing the midline. During their growth in the diencephalon, mDA axons express several axon guidance receptors. Previous work has shown that from E12.5 onward, a subset of mDA axons positioned in the ventral MFB expresses Nrp2. The Nrp2 ligand Sema3F is expressed in several brain regions surrounding the trajectory of the MFB (Kolk et al., 2009; Yamauchi et al., 2009; Torigoe et al., 2013). In *Nrp2* KO mice, the MFB is defasciculated. A similar phenotype is described in *Sema3F* KO mice and in conditional *Nrp2* KO mice in which Nrp2 is removed from mDA neurons (Kolk et al., 2009) (**Table 3**). These observations together with data showing that Sema3F is a potent axon repellent for Nrp2^+^ mDA axons *in vitro* (Hernández-Montiel et al., 2008; Kolk et al., 2009; Yamauchi et al., 2009) indicate that Sema3F acts to fasciculate mDA axons *en route* to their distant targets.

**Table 3:** Canonical axon guidance genes involved in mDA pathway development.

Axon guidance gene(s)	Species	*In vivo* observations	*In vitro* observations	Expression data	Reference
EphrinA/EphA signaling	Mice	Transgenic mice expressing EphA5ecto-Fc show 40–50% less mDA neurons innervating the striatum.			Sieber et al., 2004
*EphrinA5*	Mice^a,b^, Rats^b^	No developmental defects in mDA system^a^; 10% reduction in the number of mDA neurons innervating the striatum^b^.	mDA axon repellent^a^; mDA axon growth promotion via EphA5^b^.	mRNA expression in the ventral midbrain dorsally to TH^+^ neurons, in the thalamus, and in the striatum^a^; mRNA expression in the dorso-lateral striatum and NAc-shell^b^.	^a^Deschamps et al., 2010 ^b^Cooper et al., 2009
*EphA5*	Mice	14% reduction in the number of mDA neurons innervating the striatum.	mDA axon growth promotion via EphrinA5.	Promoter activity in developing VTA and SNc mDA neurons.	Cooper et al., 2009
*EphrinB2*	Mice		mDA axon growth inhibition of SNc neurons.	mRNA expression in the striatum: higher expression in the ventral striatum from P1 to P7, and almost no difference at E18.	Yue et al., 1999
*EphB1*	Mice	No structural defect in the mDA system^b^.		Higher mRNA levels in SNc than VTA mDA neurons from E18 to P7^a^. No promoter activity in mDA neurons, but detected in neurons of the SN reticulata^b^.	^a^Yue et al., 1999 ^b^Richards et al., 2007
*DCC*	Mice^a^, Rats^b^	Aberrant migration of a subset of mDA neurons; abnormal mDA innervation of the ventral striatum; reduced mDA innervation of the cortex, and a subset of mDA axons crosses the midline at the level of the caudal hypothalamus^a^.	Function blocking anti-DCC antibody blocks mDA axon elongation and branching induced by Netrin1^a^.	Protein expression in both SNc and VTA mDA neurons from E14 to E18^a^. In dissociated E14 mDA neurons, protein expression more abundant in SNc mDA neurons than VTA mDA neurons^b^.	^a^Xu et al., 2010 ^b^Lin et al., 2005
*Netrin1*	Mice^b-d^, Rats^a,c^, mDA neurons derived from hESC^c^	Aberrant migration of a subset of mDA neurons positioned in the reticular formation, reduced mDA innervation of the dorsal striatum. A subset of mDA axons crosses the midline at the level of the caudal hypothalamus^d^.	Promotion of mDA neurite outgrowth^a,c^, elongation^b^, attraction^c^, and branching^b^. SNc mDA axons are attracted when Netrin1 is provided at low concentrations, whereas VTA mDA axons are attracted at higher concentrations^d^.	Promotor activity in the entire mDA system from early time points, at the midline in the caudal hypothalamus, high ventro-lateral and low dorso-medial gradient in striatum^d^.	^a^Lin et al., 2005 ^b^Xu et al., 2010 ^c^Cord et al., 2010 ^d^Li et al., 2014
*Robo1*	Rats^a^, Mice^b^	KO mice show a wider MFB, with a subset of mDA axons deviating both ventrally and dorsally^b^. In *Robo1*/*Robo2* double KO mice a subset of mDA axons crosses the midline at the level of the caudal hypothalamus, mDA axons leave the MFB to grow dorsally^b^.		Expression in dissociated E14 mDA neurons^a^. Expression in some mDA neurons and by longitudinal fibers in close association with mDA axons of the MFB^b^.	^a^Lin et al., 2005 ^b^Dugan et al., 2011
*Robo2*	Rats^a^, Mice^b^	No defects in mDA system development^b^.		Expression in dissociated E14 VTA mDA neurons^a^. No expression by mDA neurons, but by longitudinal fibers in dorsal, anterior, and posterior regions of the mDA system^b^.	^a^Lin et al., 2005 ^b^Dugan et al., 2011
*Slit2*	Rats^a,b^, Mice^c,d^, mDA neurons derived from hESC^b^	A wider MFB, with a subset of mDA axons crossing the midline at the level of the caudal hypothalamus^c,d^.	Inhibition of growth and repulsion of mDA axons ^a,b,d^.	mRNA expression along the ventral midline, in the hypothalamus, and lateral thalamus^d^.	^a^Lin et al., 2005 ^b^Cord et al., 2010 ^c^Dugan et al., 2011 ^d^Bagri et al., 2002
*Sema3A*	Rats		Promotion of mDA axon growth.		Hernández-Montiel et al., 2008
*Sema3C*	Rats		Promotion of growth and attraction of mDA axons.		Hernández-Montiel et al., 2008
*Sema3F*	Rats^a,c^, Mice^a,b^	MFB defasciculation and increase in width, random orientation of mDA axons in the cortical plate^b^.	mDA axon repulsion at early time points^a-c^, axon attraction at later time points^b^.	mRNA expression in the caudal and dorsal midbrain^a,c^, and in the cortical plate^b^.	^a^Hernández-Montiel et al., 2008 ^b^Kolk et al., 2009 ^c^Yamauchi et al., 2009
*Nrp2*	Mice	Caudal growth of mDA axons^a,b^, MFB defasciculation and increase in width^a^, a subset of mDA axons crossing the midline in the caudal hypothalamus^a,c^, random orientation of mDA axons in the cortical plate^a^.	Mediates Sema3F mDA axon repulsion^a^.	Promoter activity in medial mDA neurons and in ventral MFB mDA axons^b,c^.	^a^Kolk et al., 2009 ^b^Yamauchi et al., 2009 ^c^Torigoe et al., 2013
*Sema7A*	Mice^b^, Rats^a^		Reduced axonal arborization of SNc but not VTA mDA neurons^b^.	mRNA expression in a mediolateral gradient within the developing striatum^a^.	^a^Pasterkamp et al., 2007 ^b^Pacelli et al., 2015

*Overview of *in vivo* and *in vitro* experiments reported in literature linking canonical axon guidance genes to different aspects of mDA pathway development. Expression data as reported in literature are shown.*

The caudal hypothalamus is an important intermediate target for mDA axons. In KO mice lacking Netrin1, DCC, Slit2, Slit1 and Slit2, Robo1 and Robo2, or Nrp2, a subset of mDA axons leaves the MFB at the level of the caudal hypothalamus and turns ventrally to form an abnormal axon bundle that crosses the ventral midline (Bagri et al., 2002; Xu et al., 2010; Dugan et al., 2011; Torigoe et al., 2013; Li et al., 2014) (**Figure 4C** and **Table 3**). In *Nrp2* KO mice, the use of a β-galactosidase reporter that is present downstream of the Nrp2 promoter shows that this promoter is active in the ventral part of the MFB and in the aberrantly projecting axons. Since Nrp2^+^ mDA neurons are normally positioned medially in the mDA system (Kolk et al., 2009; Yamauchi et al., 2009; Torigoe et al., 2013), these results suggest that the abnormal ventral axon projections in *Nrp2* KO mice originate from a subset of mDA neurons, most probably located in the VTA. It remains to be determined whether the aberrant ventral mDA axon bundles detected in the other KO mice also derive from specific mDA neuron subsets. Furthermore, it is unclear whether in these KO mice the abnormal projections form as a result of a lack of expression of axon repellent cues in ventral structures, e.g., such as Slit1, Slit2, and Netrin1, or of defects in other axon bundles that normally function as scaffolds for mDA axons. mDA axons are not the first axons to extend along the A-P axis of the developing brain. For example, at E13 mDA axons in the MFB interact with GAD65^+^ axons extending along the A-P axis toward the striatum. These axons might be descending fibers coming from the telencephalon and/or from the mammillary bodies of the caudal hypothalamus forming the mammillo-tegmental tract. The *in vivo* ablation of GAD65^+^ axons induces many mDA axons to turn ventrally into the hypothalamic region (Garcia-Peña et al., 2014). These data strongly suggest that (at least) a subset of mDA axons needs other pre-existing longitudinal axons as scaffolds for guidance to rostral targets.

### Innervation of the Striatum

The striatum is a major target of mDA axons. Striatal innervation by mDA axons starts around E13.5 (**Figure 4A**). *In vitro* both late embryonic and postnatal, but not early embryonic, striatal explants attract mDA axons (Gates et al., 2004). Following their entry into the striatum, mDA axons arborize extensively. Co-culturing mDA neurons with striatal neurons enhances axonal branching. The same effect is observed when culturing mDA neurons with medium conditioned by striatal neurons, indicating that secreted cues are expressed by the striatum to promote mDA branching (Manier et al., 1997). Several axon guidance cues are expressed by the striatum that may play a role in the innervation of mDA axons and the subsequent branching of these axons (**Table 3** and reviewed in Van den Heuvel and Pasterkamp, 2008; Prestoz et al., 2012). Netrin1 is an interesting candidate as it induces mDA axon attraction (Li et al., 2014), elongation, and branching (Xu et al., 2010) via DCC. Analyses of the effects of Netrin1 on SNc and VTA explants show that effects of mDA axon attraction and elongation are induced at lower Netrin1 concentrations in SNc explants as compared to VTA explants. Interestingly, SNc mDA axons do not respond to high Netrin1 concentrations (Li et al., 2014). *In vivo*, *Netrin1* expression is high in the ventro-lateral and low in the dorso-medial striatum. In *Netrin1* KO mice, SNc axons fail to innervate the dorsal striatum and accumulate in the ventral striatum (Li et al., 2014). On basis of these data it was proposed that SNc axons innervate the dorsal striatum attracted by low levels of Netrin1 expression, whereas VTA axons are directed to the ventral striatum attracted by higher Netrin1 concentration. The molecular basis for this differential sensitivity to Netrin1 of SNc and VTA mDA axons remains unknown but could include differential expression of axon guidance (co-)receptors or intracellular signaling cues.

The striatum is mainly composed of medium spiny neurons (MSNs), and displays a unique mosaic organization composed of two neuroanatomically and neurochemically distinct compartments called the matrix and patches (or striosomes) (Gerfen, 1992). Patch neurons are generated first and migrate from the lateral ganglionic eminence to the striatal mantle from E11-E12, followed by matrix neurons that start striatal mantle invasion at E13 (van der Kooy and Fishell, 1987; Mason et al., 2005). The two populations are initially intermingled in the striatal mantle, then segregate to form the matrix/patch mosaic at E18. At this age, both patch and matrix MSNs extend their dendrites almost exclusively within their respective compartments (Kawaguchi et al., 1989). The first mDA axons arrive in the developing striatum around E13, and therefore most likely receive guidance from patch MSNs. At this age, mDA innervation is sparse and scattered. From E18 onward, the entire striatum is innervated, but TH^+^ innervation is denser in striatal patches as compared to the matrix. These areas of dense TH^+^ innervation are called “dopamine islands,” and are detectable until P15, after which striatal innervation of mDA axons becomes more homogeneous (Edley and Herkenham, 1984). Initially, it was thought that in adult rat brain most mDA neurons of the ventral SNc were arborizing in patches, while dorsal SNc mDA axons were innervating the matrix compartment (Prensa and Parent, 2001). However, more recently it has been demonstrated that in adult mice both ventral and dorsal SNc mDA neurons innervate both striatal compartments (Matsuda et al., 2009).

Depletion of mDA neurons at early developmental timepoints does not cause changes in striatal patch/matrix organization. This indicates that aggregation of patch MSNs is independent from mDA innervation (Snyder-Keller, 1991). In contrast, the formation of ‘dopamine islands’ strictly relies on correct striatal development. In *Ctip2* KO mice and *Notch1* KO mice, where patch/matrix organization is lost and patches are not formed, dopaminergic innervation is correct at E15 (before aggregation of patch MSNs occurs), but disorganized and diffuse at P0 without discernable ‘dopamine islands’ (Mason et al., 2005; Arlotta et al., 2008). These results suggest that patch MSNs express molecular cues that induce the aggregation of a subset of mDA axons to form ‘dopamine islands.’ Interesting candidates for such a role are Ephs and ephrins. For example, the EphA4 receptor and its ligand ephrinA5 are expressed by matrix and patch MSNs, respectively, and are required for the correct development of striatal compartments (Passante et al., 2008). Other Ephs are also enriched in striatal patches, e.g., EphA7 and EphA8 (Allen Brain Atlas). The precise function of ‘dopamine islands’ during development remains to be determined, whether their transient appearance is necessary for the development of the postnatal brain, and which guidance molecules induce mDA axons to aggregate in the patch compartment.

### Innervation of the Medial Prefrontal Cortex

VTA mDA axons follow two trajectories to reach the mPFC: (1) a subset of axons exits from the MFB at the point where most mdDA axons enter the striatum and then extends rostrally to move into the PFC just caudal of the olfactory bulb; and (2) another subset of VTA mDA extends through the striatum, crosses the external capsule and innervates the mPFC. Within the mPFC, the first mDA axons are detected in the subplate (SP) and the marginal zone (MZ) around E15. At E16.5, the innervation of these regions increases, while the cortical plate (CP) remains devoid of mDA axons. After a waiting period of 2 days, mDA axons invade the CP from the SP, following a radial path (Kolk et al., 2009). Such a waiting period for mesocortical mDA projections is observed also in human PFC (Verney, 1999).

*In vitro* mPFC explants release diffusible molecules that induce mDA VTA subset-specific axon outgrowth. From E14 onward, rostral VTA explants are attracted by mPFC, whereas caudal VTA explants are repelled (Kolk et al., 2009). This observation strongly suggests that rostral VTA mDA neurons, but not caudal VTA mDA neurons, constitute the VTA cell subtype that establish connections with the mPFC. Sema3F is expressed in the CP and exerts an interesting bifunctional guidance effect on rostral VTA explants: at E12.5, Sema3F is a strong repellent, while at E14.5 becomes an attractant. Both effects are mediated by the Nrp2 receptor. Despite the presence of several mDA axonal defects along the trajectory of mDA axons (caudal growth into the midbrain, MFB defasciculation, and hypothalamic innervation), many mDA axons reach the mPFC. Here, chemoattraction mediated by Sema3F and Nrp2 is required to orient mDA axons projections in the CP (Kolk et al., 2009). The bifunctional effect of Sema3F is an important molecular mechanism that allows one molecule to exert distinct effects in different spatio-temporal conditions. As we discussed previously, Sema3F acts first as a repellent for mDA axons in the midbrain, directing them toward the diencephalon. Then, when mDA axons approach the mPFC, Sema3F becomes an attractant to promote the correct innervation of the CP. Although, it has been demonstrated that both the attractant and repellent effects of Sema3F are mediated by Nrp2, further work is needed to dissect how a single receptor can transduce two opposite biological effects.

### Innervation of the Habenula

**FIGURE 5:**
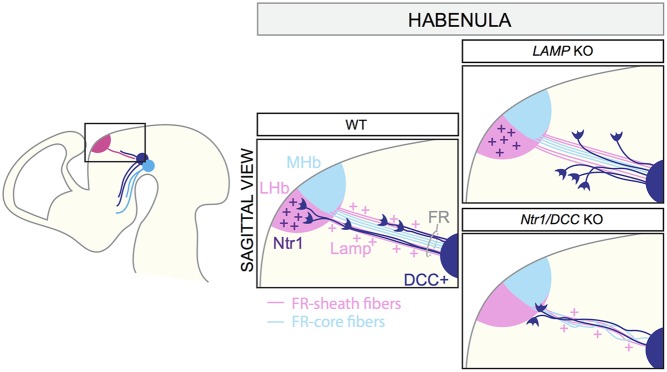
Axon–axon interactions during the formation of dopaminergic input of the habenula. MHb and LHb projections grow toward the ventral midbrain forming the fasciculus retroflexus (FR). LHb fibers form the FR-sheath, while MHb axons are confined inside forming the FR-core. Habenular axons of the sheath express the adhesion molecule LAMP, which allows the physical interaction of mDA axons and FR-sheath fibers important for mDA axon guidance. Once mDA axons arrive at the edge of the LHb nucleus, Netrin1 expressed by the LHb neurons attracts mDA axons. DCC expressed by mDA axons mediates Netrin1 signaling. In *LAMP* KO mice, mDA axons are detached from the FR and, as a consequence, they cannot reach the LHb nucleus. In both *Netrin1* and *DCC* KO brains, mDA axons no longer innervate the LHb, but instead stall at its ventral border. LHb, lateral habenula; MHb, medial habenula; FR, fasciculus retroflexus.

The habenula is positioned in the dorso-medial diencephalon and is composed of two main subdomains: the lateral (LHb) and the medial habenula (MHb). The MHb projects primarily to the interpeduncular nucleus, whereas the LHb innervates monoaminergic nuclei, including the mDA system (Hikosaka et al., 2008). Efferent habenular axons fasciculate together to form the fasciculus retroflexus (FR), which has an outer sheath of LHb axons surrounding a core of MHb axons (Bianco and Wilson, 2009). VTA mDA axons projecting to the LHb are confined to the sheath domain of the FR (Schmidt et al., 2014; **Figure 5**).

VTA mDA axons start growing toward the habenula around E12.5 and arrive at this structure by E13.5. At E16.5, mDA axons are detectable in the LHb, but not in the MHb. The first phase of mDA axon navigation relies on the FR that is used as a scaffold by mDA axons. *In vivo* genetic ablation of the habenula and blockage of habenular axons *in vitro* prevent mDA axon growth toward the habenula. The physical interaction between mDA axons and axons of the sheath is required for the correct development of the system, and limbic-system associated membrane protein (LAMP) is an important mediator of this process. Reduced expression or lack of LAMP from LHb-sheath axons, or blockage of LAMP function by anti-LAMP antibodies induces the detachment of TH^+^ axons from the FR, impeding their growth to the LHb (Schmidt et al., 2014). Once mDA axons arrive at the habenula, the innervation of the LHb is mediated by Netrin1/DCC signaling. Netrin1 is expressed by the LHb, but not the MHb, and is a potent attractant for the DCC^+^ mDA axons that are approaching the lateral nucleus. In both *Netrin1* and *DCC* KO mice, mDA axons no longer innervate the LHb, but instead stall at its ventral border (Schmidt et al., 2014). In conclusion, these data show that the LHb determines its own dopaminergic afferents by projecting axons toward the VTA. mDA axons are collected and guided by habenular axons of FR-sheath, relying on a mechanism of reciprocal axon–axon interactions. This process cooperates with a mechanism of LHb-specific chemoattraction, which controls the target entry of the LHb (**Figure 5**).

### Subset-Specific Transcription Factors Control Axon Guidance Gene Expression

As described in this section, during brain development, the expression of different axon guidance receptors allows subsets of mDA axons to respond to specific environmental cues that steer them to the correct target. The expression of different guidance receptors derives from the activation of specific developmental programs that differ between mDA neuron subsets. Many studies have analyzed which transcription factors specifically characterize SNc and VTA mDA neurons, but only a few studies have focused on the expression of axon guidance receptors induced by subset-specific transcription factors. Interesting examples are the transcription factors Otx2 and Sox6, expressed by VTA and SNc mDA neurons, respectively (Panman et al., 2014). *In vitro* overexpression of Otx2 in mDA neurons increases the expression of the guidance receptors Nrp1 and Nrp2, whereas PlxnC1 and EphB3 levels are unaffected. Downregulation of Otx2 causes the opposite effect. This indicates that transcription of *Nrp1* and *Nrp2* in VTA mDA neurons relies on the subset-specific transcription factor Otx2 (Chung et al., 2010). As we discussed, Nrp2 is an important receptor required during several stages of VTA mDA axon navigation (e.g., mDA axon orientation in the midbrain, fasciculation of the MBF, and mPFC innervation). Sox6 is necessary for EphA5 expression in SNc mDA neurons (Panman et al., 2014). In *Sox6* KO mice, SNc mDA neurons lack EphA5 expression (Panman et al., 2014), which is required for the correct innervation of the striatum (Cooper et al., 2009). However, in contrast to the subset-specific expression of Nrp2, the EphA5 promoter is active in both SNc and VTA mDA neurons (Cooper et al., 2009). This suggests then that the expression of EphA5 in VTA mDA neurons is regulated by another transcription factor. Future studies are needed to further unravel the transcription factor networks that control subset-specific axon guidance cue expression.

## Future Directions

Because of their diverse functional roles and their implication in disease, mDA neurons have been studied extensively. Many studies have recently focused on dissecting the molecular programs that dictate the formation of mDA connectivity. This has led to the identification of an increasing number of molecular cues that control the migration of embryonic mDA neurons or the guidance of their axons. Furthermore, approaches such as single cell-omics have begun to provide insight into the different neuronal subsets that comprise the mDA system. More studies focused on further understanding the heterogeneity of mDA neurons are needed for several reasons: (1) SNc mDA neurons are more prone to degeneration in Parkinson’s disease than VTA neurons (Albin et al., 1989). The identification of molecular profiles that correlate with different mDA neurons subsets may increase our understanding of why certain neurons are more vulnerable to degenerate than others. (2) Understanding the molecular features of different mDA subsets may improve the generation and the characterization of iPSC-derived mDA neurons subsets. This will help to obtain better *in vitro* models of human mDA cell-types and more specific mDA neurons to transplant into Parkinson’s disease patients. (3) New genetic markers selective for mDA subsets can be used to generate Cre or Flp mouse lines to target mDA cell types. These new mouse lines would allow the expression of fluorescent reporter proteins for the visualization of mDA neuronal subsets, to conditionally knock-out genes and study their functions, and to perform optogenetic analyses on molecularly homogeneous mDA neurons. In particular, *in vivo* florescent labeling of mDA neuron subsets will allow researchers to study migration and axon guidance of each neuronal subset individually, to isolate mDA subsets, and to determine their precise molecular profile. In addition, this approach will allow the analysis of structural alterations of mDA neuron subsets in mice lacking axon guidance genes involved in mDA system wiring, to specifically link the function of axon guidance genes to mDA neuronal cell types.

Despite recent progress, many questions remain. For example, in the adult brain, mDA neurons receive afferents from many different brain areas, but the developmental programs involved in establishing mDA afferent connections remain largely unidentified. Furthermore, while many cues are now known to affect mDA axons, the structure and development of mDA dendrites remains largely unexplored. Technological advances such as light-sheet microscopy and single-cell omics approaches will help to provide insight into these and other questions.

A better understanding of mDA system development is also essential from a clinical perspective. mDA neurons of the VTA have been implicated in disorders such as drug addiction, depression, and schizophrenia (reviewed in Morales and Pickel, 2012; Walsh and Han, 2014), and evidence indicates that developmental and/or adult structural changes of neuronal networks may in part underline the pathogenesis of these disorders (Robinson and Kolb, 2004). In particular for drug addiction-behavior, it has been shown that the expression of axon guidance genes changes in the mDA system after a long administration of drugs, and drug-induced behaviors can be altered upon genetic manipulation of axon guidance genes (Sieber et al., 2004; Flores et al., 2005; Auger et al., 2013; Pokinko et al., 2015). Furthermore, genome-wide association studies and gene expression profiling have linked axon guidance proteins to Parkinson’s disease (reviewed in Van Battum et al., 2015). Due to their roles in mDA system development, changes in the expression or function of axon guidance genes may lead to defects in the formation or maintenance of mDA neuron connectivity and function. Although axon guidance events are crucial for the correct development of mDA nigrostriatal and mesocorticolimbic pathways, their precise role during the pathogenesis of mDA system-related diseases has not been established. Further studies on the role of axon guidance molecules during the development and plasticity of mDA networks are required to better understand their potential contribution to diseases. Understanding how the dopamine system develops will also aid the development of more effective of cell-replacement strategies in Parkinson’s disease patients. The molecular ingredients required to build a functional mesostriatal pathway could be applied to improve the integration of transplanted mDA neurons into the degenerating dopamine system.

## Author Contributions

SB designed Figures. SB and RJP wrote the manuscript.

## Conflict of Interest Statement

The authors declare that the research was conducted in the absence of any commercial or financial relationships that could be construed as a potential conflict of interest.
